# Environmentally Sustainable Preparation of Oleuropein and Its Dialdehydic Derivatives in a Simple Pharmaceutical Formulation

**DOI:** 10.1002/cssc.202402252

**Published:** 2025-01-07

**Authors:** Monica Nardi, Sonia Bonacci, Marialaura Frisina, Manuela Oliverio, Rosa Scarpelli, Antonio Procopio

**Affiliations:** ^1^ Department of Health Science – AGreen Food Research Center University Magna Græcia of Catanzaro Viale Europa 88100 Catanzaro Italy

**Keywords:** Oleuropein, Oleacein, EVOO, Deep Eutectic Solvents

## Abstract

In this paper, Krapcho's one‐step decarbomethoxylation of oleuropein in DES is reported. Oleuropein used as reagent was extracted with water from olive leaves, widely available and inexpensive waste from the olive oil production chain. The reaction has been carried out in a series of ChCl‐based DES of increasing acidity, with or without the addition of an amount of water, under microwave and conventional heating. The antioxidant power of the best reaction mixture, both in terms of biocompatibility of the medium and conversion of the starting material, was measured and compared with a natural phenolic mixture coming from EVOO. The reported results indicate that the formulation deriving by the Krapcho's one‐step decarbomethoxylation of oleuropein in ChCl:Citric acid (1 : 1) DES can provide a ready‐to‐use “phenolic complex“ with oxygen scavenging power similar to a mixture of phenols extracted from an EVOO that meets the requirements of EFSA health claim.

## Graphical abstract

A “phenolic complex“ with oxygen scavenging power similar to a mixture of phenols extracted from an EVOO was obtained by the one‐step Krapcho's decarboxylation of oleuropein in ChCl:citric acid 1 : 1 DES. The mixture is a potential ready‐to‐use active formulation able to emulate the antioxidant power of EVOO.

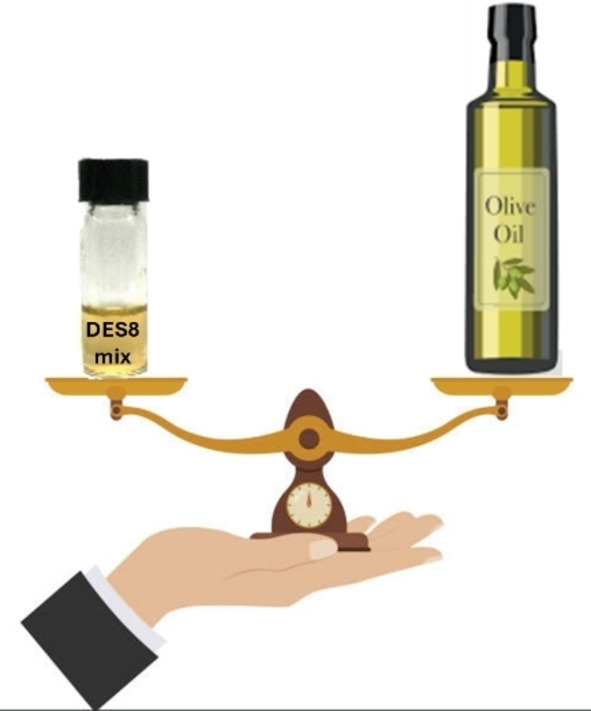

## Introduction

**Figure 1:**
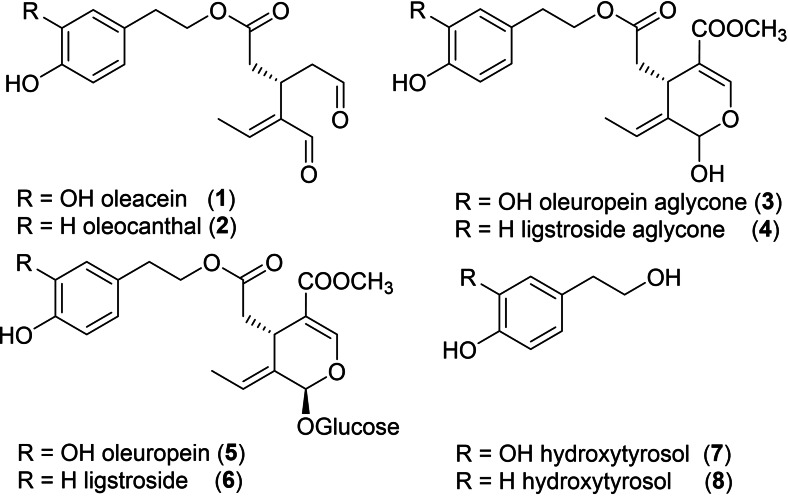
Main bioactive olive secoiridoids.

The crucial role of Extra Virgin Olive Oil (EVOO) in determining the beneficial effects of the Mediterranean diet on human health has long been associated with its phenolic constituents that are also responsible for some organoleptic characteristics of the high‐quality EVOOs (i. e., bitterness, pungency, and astringency).^[1]^ The most known phenolic compounds of EVOO are the secoiridoid derivatives oleacein (3,4‐DHPEA‐EDA) **1**, oleocanthal (*p*‐HPEA‐EDA) **2**, monoaldehydic form of oleuropein aglycone (3,4‐DHPEA‐EA) **3**, and monoaldehydic form of ligstroside aglycone (*p*‐HPEA‐EA) **4** which come from the secoiridoid glucosides oleuropein **5** and ligstroside **6**, enzymatically hydrolysed during the pressing of the olive fruit to obtain EVOO (Figure 1).[^2^, ^3^]

Due to their hydrophilic nature, these dialdehydic phenols (**1**–**4**) are only partially transferred from olive fruits and paste to the oil, generally up to 500 mg/L,^[4]^ with the remaining amount lost in the olive mill wastewater (~53 %) and in the pomace (~45 %).^[5]^ Thus, oleacein **1** and oleocanthal **2** are the principal aldehydic constituents of EVOO, structurally very similar to each other differing only by their aromatic moiety, hydroxytyrosol and tyrosol respectively. In recent years, these two dialdehydes have raised a wide scientific interest owing to their biological activities (*e. g*., anti‐atherogenic, anti‐hepatotoxic, anti‐inflammatory, antioxidant, anti‐tumoral, anti‐viral, analgesic, hypoglycaemic, cardioprotective and neuroprotective)[^6^, ^7^, ^8^, ^9^, ^10^, ^11^, ^12^, ^13^, ^14^, ^15^, ^16^, ^17^] despite their low availability from both natural and synthetic sources. More than a decade ago, the beneficial effect of these compounds was recognized by the European Food Safety Authority (EFSA) which established that “*olive oil polyphenols contribute to the protection of blood lipids from oxidative stress”*, specifying that “*The claim may be used only for olive oil which contains at least 5 mg of hydroxytyrosol and its derivatives (e. g. oleuropein complex and tyrosol) per 20 g of olive oil. In order to bear the claim information shall be given to the consumer that the beneficial effect is obtained with a daily intake of 20 g of olive oil*.”^[18]^ Interestingly, although the health claim refers indifferently to olive oil polyphenols containing in their structure hydroxytyrosol or tyrosol, there is less information on the biological activity of oleacein (3,4‐DHPEA‐EDA) (**1**) and monoaldehydic form of oleuropein aglycone (3,4‐DHPEA‐EA) (**3**) which derives from the secoiridoid oleuropein,^[19]^ abundant and easily accessible from *O. europaea* (up to 10 % in dried leaves), than oleocanthal (2) which derives from the rarer ligstroside.^[20]^ Oleacein (**1**) was reported together with oleocanthal (**2**) for the first time in 1993^[21]^ and it has been found in EVOO in larger amounts then more its hydrophilic precursors oleuropein and oleuropein aglycone (**3**).[^22^, ^23^] However, the difficult and expensive extraction and purification of oleacein from EVOO still prevents obtaining large quantities of the pure compound at affordable prices, pushing actually research towards the development of efficient semi‐synthetic strategies. Therefore, several methodologies have been proposed in the last decade to obtain oleacein **1** from the demethyloleuropein scarcely present in olive tissues^[25]^ or the widely available oleuropein.^[3, 25]^ In the latter, unfortunately, Krapcho's one‐step decarbomethoxylation of oleuropein^[26]^ gives oleacein up to 80 % yield but leaves the product in solutions from which extraction is always very problematic (DMSO or water).

On the other hand, even fewer preparation procedures have been reported for oleuropein aglycone,^[3, 25]^ which is naturally highly unstable and rearranges in different isomers.^[3]^

Natural deep eutectic solvent (NADES) is a term introduced by Choi et al^[29]^ to describe a subclass of deep eutectic solvents (DES), firstly described by Abbott et al.,^[30]^ recognized as GRAS (Generally Recognized as Safe) cheap, and sustainable being made up of components that are present in our daily food. NADES are liquids obtained by combining molecules abundantly occurring in nature, having a lower melting point than either of the components^[31]^ with the ability to solubilize, store or transport water‐insoluble metabolites in cells and living organisms.^[32]^ For all these reasons, nowadays NADES are widely used in plant‐extract production for direct use in pharmaceutical and cosmetic formulations as well as food‐related applications.[^33^, ^34^, ^35^, ^36^] Notably, in a recent work, the French company Naturex proposed the relatively benign betaine/lactic acid NADES, to create a range of plant extracts with antioxidant properties greater than the corresponding alcoholic solutions.^[37]^ In the last decade, some NADES based on choline chloride and some natural molecules (xylitol, 1,2‐propanediol, citric acid, lactic acid, etc.) have been successfully employed as extraction solvents of phenolic compounds from olive oil by‐products.[^38^, ^39^, ^40^, ^41^, ^42^] In many cases, the ability of phenolic compounds to act as HBD and therefore to compete with alcoholic or acidic HBDs in the interaction with the chloride anion has been highlighted,^[40]^ suggesting the possibility of carrying out in such NADES the eco‐sustainable chemical manipulation of the natural EVOO phenols. Considering that in our previous attempt to obtain oleacein, we realized that, in addition to Krapcho decarboxylation, sugar hydrolysis always occurs also with the formation of aglycone **3**, giving a mixture of oleacein, oleuropein aglycone and oleuropein that resembles most of the qualitative composition of olive oil, the main goal of our work is to realize an ecological and simple method to obtain a formulation that mimics the mixture of phenols of extra virgin olive oil in one step from the readily available natural oleuropein, allowing the direct production of a pharmaceutical formulation in NADES capable of maintaining the properties recognized by EFSA to EVOO.

## Results and Discussion

### Synthesis

**Scheme 1:**
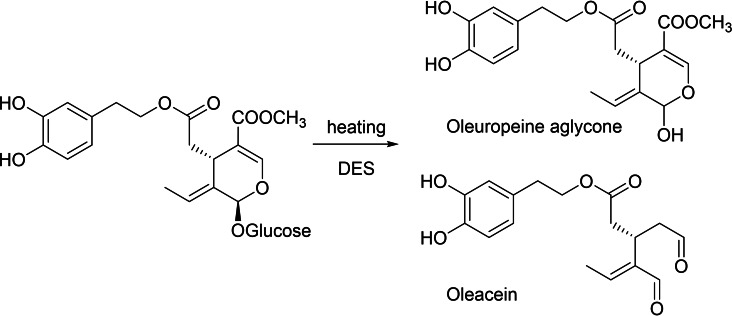
Krapcho's one‐step decarbomethoxylation in DES.

Considering the recently reported role that ChCl plays as a promoter for the direct amidation of carboxylic acids and in situ amine protection,[^43^, ^44^] we decided to test the possibility of simply using DES ChCl and water to trigger Krapcho's one‐step decarbomethoxylation reaction that begins with the aglycone hydrolysis step (Scheme 1).

Subsequently, the ChCl‐based DESs reported in Table 1 were tested with increasing acidity with or without the addition of 10 % water, which was enough to reduce the viscosity of the NADES and increase the conductivity while maintaining the original DES structure.^[45]^ As a term of comparison, some betaine DESs were also used which proved to be particularly suitable for the extraction of active ingredients from plant matrices.^[37]^ All the DES were prepared by heating the components to 80 °C under agitation until a homogeneous liquid was formed and then, where required, by adding water 10 % v/v (DES3w‐DES9w).

**Table 1:** Compositions and abbreviations for the prepared DESs.^[a]^

Acronym	Composition	Ratio
DES1	ChCl: Water	1 : 1
DES2	betaine: water	1 : 3.3
DES3	ChCl: urea	1 : 1
DES4^[b]^	betaine: ethylene glycol	1 : 3.3
DES5	ChCl: p‐TSA	1 : 3
DES6	ChCl: lactic acid	1 : 1
DES7	ChCl:ascorbic acid	1 : 1
DES8	ChCl:citric acid	1 : 1
DES9	ChCl:citric acid	2 : 1
DES3w^[c]^	ChCl: urea	1 : 1
DES4w^[c]^	betaine: ethylene glycol	1 : 3.3
DES5w^[c]^	ChCl: p‐TSA	1 : 3
DES6w^[c]^	ChCl: lactic acid	1 : 1
DES7w^[c]^	ChCl:ascorbic acid	1 : 1
DES8w^[c]^	ChCl:citric acid	1 : 1
DES9w^[c]^	ChCl:citric acid	2 : 1

^[a]^ The DES components under stirring for 2 h at 60 °C;. ^[b]^ Citric acid (1 %). ^[c]^ 10 % of water was added.

Oleuropein was extracted as reported by Procopio et al.^[46]^ from olive leaves of Coratina cultivar of *Olea europaea* L. which are considered waste from the olive oil production chain, and purity was determined by RP‐HPLC and HRMS‐ESI and ^1^H‐NMR data compared with data reported in the literature.

The Krapcho's one‐step decarbomethoxylation reaction reported in Scheme 1 was performed by heating the oleuropein:DES mixture. 1 : 1 by microwave assisted or conventional heating (80 °C) for 10–60 minutes, then the reaction mixture was analyzed by HPLC. Results were compared with those reported in Ref 27b.

**Figure 2:**
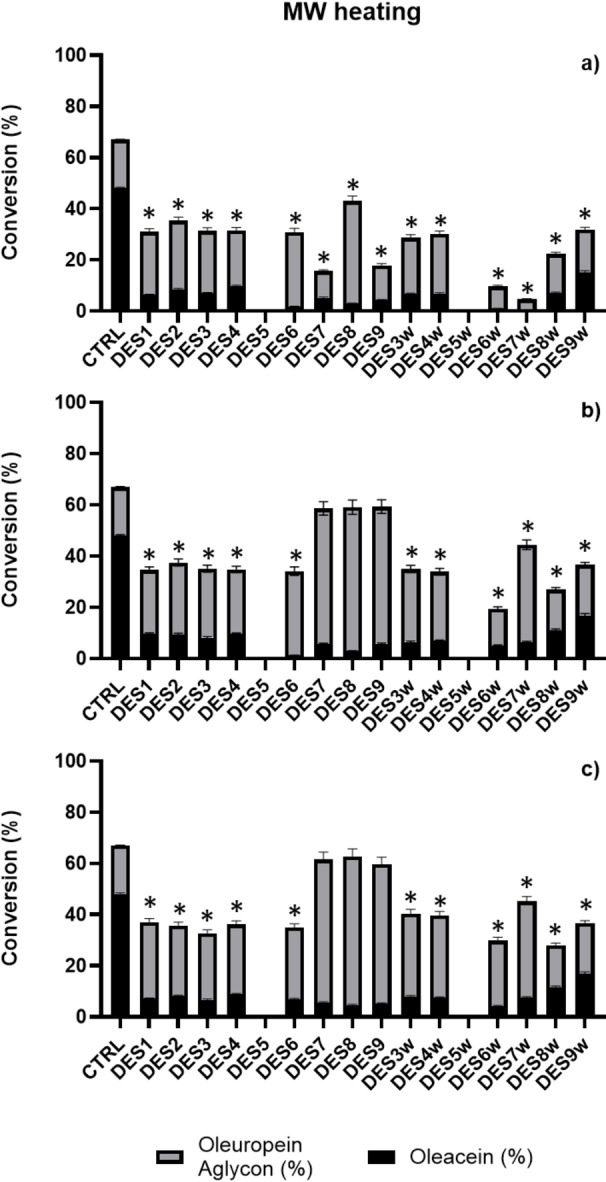
MW‐assisted (149 °C) Krapcho's one‐step decarbomethoxylation in DES after 10 min (panel a), 30 min. (panel b) and 60 min. (panel c). Conversion was evaluated by HPLC and compared with the results reported in Ref. 27b as positive control (CTRL). Data are expressed as mean±SD of three independent observations. Two‐way ANOVA followed by Dunnett test was used for the analysis of variance. *p<0,001.

**Figure 3:**
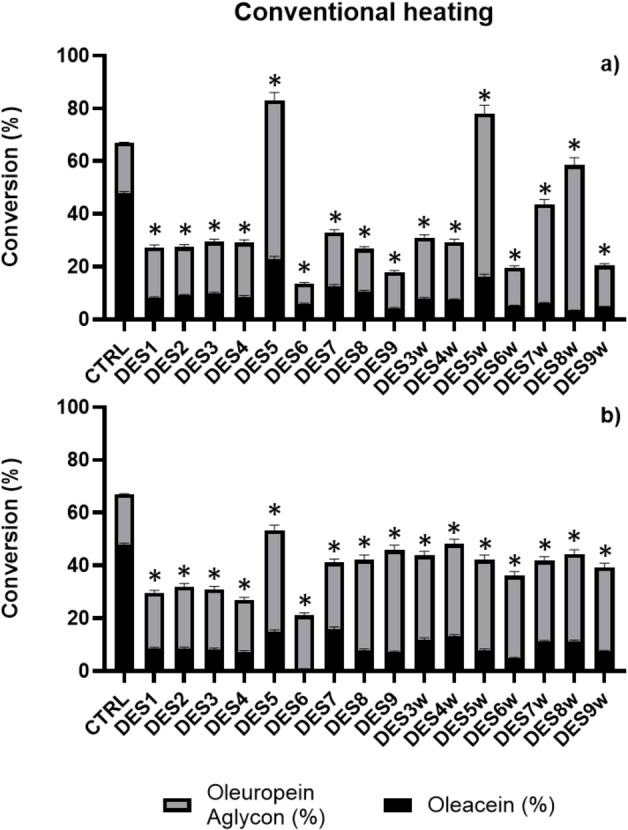
Krapcho's one‐step decarbomethoxylation in DES under conventional heating (80 °C) after both 30 min (panel A) and 60 min (panel B) of reaction. Conversion was evaluated by HPLC.and compared with the results reported in Ref. 27b as positive control (CTRL). Data are expressed as mean±SD of three independent observations. Two‐way ANOVA followed by Dunnett test was used for the analysis of variance. *p<0,001.

Firstly, we performed the experiments with the selected DESs under the same conditions as in our previous experience of Krapcho decarbomethoxylation of oleuropein to obtain MW‐assisted oleacein at 149 °C (Figure 2 and Table S1in S.I.). After only 10 min of reaction time, the best result was recorded for DES8 with a conversion of the oleuropein starting material of 48 % and a yield of just under 3 % in oleacein and 40 % in oleuropein aglycone. The addition of 10 % water to the DES used in the experiment led to a general worsening of the oleuropein conversion yields with the best performance being achieved by DES9w, the only one to benefit from the addition of water by nearly doubling the % conversion of the starting material, giving almost equivalent amounts of oleacein and aglycone (15.0 % and 16.9 % respectively) which is the best result obtained in this first set of experiments for oleacein (Figure 2 and Table S1 in Supporting Information). Extending the reaction times up to 30 min., under the same MW conditions, led to a general increase in the oleuropein conversion yields up to almost 60 % for DES9, DES8 and DES7 in that order, although with a poor oleacein yield, since almost all the starting material was transformed into oleuropein aglycone (the highest result was still for DES8 with 56 %). Also in this case, the addition of 10 % water led to a general worsening of the conversion yields, with the best performance being achieved by DES7w with just over 44 % conversion of the starting material, but only 6.5 % of oleacein (Figure 2 and Table S1 in Supporting Information). In this second set of experiments, the best result regarding the synthesis of oleacein is the one observed in DES9w with a conversion of 36.6 % of oleuropein, which gives 16.7 % of oleacein and just under 20 % of aglycone. Given the positive trend in conversion percentages with increasing reaction times, we performed the same experiments, extending the reaction times to 60 min. Once again, we observed a general increase in the conversion percentages of the starting oleuropein, with better results again for DES7, DES8 and DES9 with a maximum conversion of 62.8 % for DES8 and a yield of oleacein and oleuropein aglycone of 4.7 and 58.1 % respectively. Also in this case, oleacein yields greater than 8.8 % (DES 4) were not achieved for any of the DES systems used, and oleuropein aglycone still remaining the main conversion product of oleuropein. The addition of 10 % water, again recorded a general decrease in conversion yields, albeit with a slight increase in oleacein yields (Figure 2 and Table S1 in Supporting Information). Although DES7w still recorded the highest percentage of starting material conversion (45.2 %), the best yield in oleacein was obtained again for DES9w with 16.7 %, accompanied by 19.9 % of aglycone, and a total conversion yield of 36.7 %. Increasing the reaction times beyond 60 minutes led to a general decrease in conversions evidenced by the presence of degradation products (data not shown). Summarizing the experiments conducted with the promotion of MW, it is highlighted that the DESs 7–9 give the best results in terms of percentage of conversion of the starting oleuropein and that the addition of 10 % of water does not improve the reaction performance in any of the six DES systems investigated (Figure 2 and Table S1 in Supporting Information). However, the addition of water improves the conversion of oleuropein into the final product oleacein although still with the unsatisfactory maximum yield of 16.75 % for DES9w. It should be noted that, in the case of the more acidic DES5 based on p‐toluensulfonic acid (p‐TSA) (ChCl: p‐TSA 1 : 3), the activation with MW was always too aggressive, giving the complete carbonization of the reaction mixture even after only a few minutes (Figure 2 and Table S1 in Supporting Information). For this reason, we decided to perform the Krapho decarbomethoxylation reaction of oleuropein in the p‐TSA DES systems, but by heating the reaction mixture with conventional means (Figure 3 and Table S1 in Supporting Information). The first reaction run, carried out at 80 °C for 30 min, revealed a general decrease in the conversion percentages of the starting material, but with a surprising 83.0 % conversion of oleuropein and a yield of 22.8 and 60.3 % of oleacein and aglycone, respectively, in the ChCl:p‐TSA 1 : 3 system (DES5). On the contrary, in the DESs 7–9, among the best in the case of MW‐activated reactions (~60 %), an oleuropein conversion of only 33 % is observed (in the case of DES7). The addition of 10 % of water to the DES systems considered seems to slightly improve the conversion yields, especially for the DESs 7–9w up to 58.1 % for DES8w. The highest conversion yield of starting oleuropein, however, is still recorded for DES5w (78.1 %, 16.28 % oleacein and 61.85 % oleuropein aglycone) although lower than the experiment without the addition of 10 % water (Figure 3 and Table S1 in Supporting Information). Increasing the reaction times to 60 min at 80 °C, in the attempt to further improve the conversion yields, did not lead to the desired result, at least in the best performing case of DES5, for which a drop in yield of up to 53.4 % was observed with 14.8 % oleacein and 38.6 % oleuropein aglycone. However, a longer reaction time led to a general increase in the conversion yields of the starting material in the other DES systems without ever reaching, however, the percentages observed in DES5 with the best results registered for DES8 with 48.2 % conversion and a yield of oleacein and oleuropein aglycone of 13.1 and 35.04 % respectively. Again, no significant improvements were recorded with the addition of 10 % water to the DES systems considered, with a general flattening of the conversion yields of the starting material around 40.0 % (maximum for DES8w 44.3 % with 11.2 of oleacein and 33.1 % of aglycone). From the analysis of the data above discussed, it come out that the best result (22.8 % oleacein, 60.2 % aglycone, 17 % oleuropein) was obtained in DES5 by conventional heating at 80 °C for 30 min (Figure 3 and Table S1in S.I.).

However, the best results obtained for Krapcho's one‐step decarbomethoxylation of oleuropein in a biocompatible DES are the ones reported for DES7 and DES8, formed by ChCl/ascorbic acid and ChCl/citric acid (1 : 1 w/w) respectively. In these two DES, after 60 min at 149 °C, Krapcho's one‐step decarbomethoxylation of oleuropein promoted by MW gave more than 60 % conversion with the percentages of oleacein and oleuropein aglycone reported in Table S1 (see Supporting Information).

### Formulation and Characterization of Synthetic Extracts

The natural mixture of phenolic derivatives, contained in an EVOO that meets the requirements of the EFSA claim, is more complex than that obtained by our synthetic procedure starting from oleuropein alone. For this reason, we decided to compare the antioxidant power of a natural EVOO with the mix of phenolic derivatives obtained by applying our Krapcho's one‐step decarbomethoxylation in DESs on oleuropein extracted from *Coratina* olive leaves, a waste product of the olive oil production chain.

Therefore, we evaluated the oxygen radical absorbance capacity (ORAC) a standardized test usually applied for the chemical measurement of the food and beverage antioxidant activity, by classical radical scavenging reaction. The chemical mechanism involved is the radical chain breaking by H atom transfer.[^47^, ^48^, ^49^] The method provides a controllable source of peroxyl radicals simulating their reactions with lipids and the antioxidant inhibitions in both food and physiological systems.

The test was performed on five samples, namely EVOO natural extract (NE), obtained by hydroalcoholic extraction of a sample of EVOO produced in 2024 from *Carolea* olives and meeting the requirements of the EFSA claim^[50]^ (see Experimental section for details), the phenol mixture coming from DES5 (SE1) and DES8 (SE2) recovered by passing the reaction solution through a column packed with Amberlyst A21 free‐base adsorbent resin, the phenol crude reaction mixture obtained in DES8 (DES8_mix_) and blank DES8 as negative control (DES8) (Table 2). The selected mixtures were our best synthetic result, and our best synthetic result obtained in a biocompatible DES, respectively. Indeed, DES8 formed by ChCl/citric acid (1 : 1 w/w) can be considered safe for the human health,[^51^, ^52^] and we hypothesised that the phenol mixture obtained in this DES could be directly used in cosmetic and nutraceutical preparations, without any further purification.

**Table 2:** Comparison in the ORAC_FL_ values between selected reaction mixtures and natural EVOO extract.

Sample	Concentration^[a]^	ORAC_FL_^[b]^
NE	5	10022±159
SE1	5	10729±199
SE2	5	4051±890
DES8mix	5	3217±121 3072±135^[c]^
DES8	5	–

^[a]^ mg/L; ^[b]^ Data expressed as means ±SEM of three independent observations (μmol Trolox/gr of dry matter); ^[c]^ ORAC test performed on DES8 mix after six months of storage at r.t.

Antioxidant capacity of all samples (10 mg/L) was measured using fluorescein (FL) as fluorescent probe: the inhibition of the peroxyl‐radical induced oxidation of FL, initiated by thermal decomposition of 2,2′‐azobis(2‐amidino‐propane)dihydrochloride (AAPH), was evaluated as validated in literature^[48]^ (see pag S8–S9 in Supporting information).

As reported in Table 2, the antioxidant power of SE1 obtained by the Krapcho's one‐step decarbomethoxylation of oleuropein in DES5 is comparable to that of NE, a mixture of phenols naturally extracted from EVOO. This confirms, at least about the oxygen scavenging power, that the mixture of phenols obtained from oleuropein alone is able to emulate the performance of the more complex mixture of phenols extracted from an EVOO that meets the requirements of the EFSA request.^[48]^ Instead, a lower antioxidant power was recorded for SE2, the mixture of phenols obtained from oleuropein subjected to Kracpcho decarboxylation in the more biocompatible, but less acidic, DES8. However, considering the greater biocompatibility of DES8, the result obtained can still be considered satisfactory, especially because it would offer the possibility of using the mix of phenols obtained from the reaction DES8 without further purification. In this regard, the ORAC test was performed on DES8mix, the reaction mixture as it is, and on blank DES8, to rule out the possibility that it had oxygen scavenging activity. The antioxidant power of DES8mix is approximately 20 % lower than that of the same mix of phenols separated from the reaction mixture in DES8, which is surprising considering that it is constituted by 50 % of the inactive DES8 (see Table 2). This small difference in the ORAC test could be attributed to the interactions between phenols and the components of the DES mixture.[^53^, ^54^] However, the antioxidant power measured by the ORAC test allows us to consider that only 3 grams of DES8mix would correspond to an amount of phenols possessing the same oxygen scavenging power of 20 gr of EVOO, according to EFSA claim. Noteworthy, while an EVOO that meets the requirements of the EFSA request, progressively loses its phenol content over time,^[50]^ the mixture of phenols obtained from Krapcho's one‐step decarbomethoxylation of oleuropein obtained from waste olive leaves, maintains its total phenol content and the respective ratios almost unchanged, as shown by the ORAC test repeated on the DES8mix after 6 months of storage at r.t. (Table 2) and the HPLC analysis (Figure S2 and S3 in Supporting information).

## Conclusions

In this paper, Krapcho's one‐step decarbomethoxylation of oleuropein in DES is reported. The starting material was extracted with water from olive leaves, widely available and inexpensive waste from the olive oil production chain. The reaction has been carried out in a series of ChCl‐based DES of increasing acidity with or without the addition of an amount of water thus maintain the DES structure. The reaction was activated by microwave or conventional heating giving its best result, in terms of conversion of the starting material, into the most acidic DES composed of ChCl and *p*‐toluenesulfonic acid 1 : 1 (DES5) without addition of water. However, among the less acidic NADES, the best performances was recorded for the DES composed by ChCl and citric acid 1 : 1, always without addition of water (DES8). The antioxidant power of the purified mixtures of phenols obtained from Krapcho's decarbomethoxylation of oleuropein in DES5 and DES8 (SE1 and SE2) together with the most biocompatible reaction mixture itself (DES8mix) were measured and compared with a natural phenolic mixture coming from EVOO (NE). The reported results indicate that oleuropein alone can provide a “phenolic complex“ with oxygen scavenging power similar to a mixture of phenols extracted from an EVOO that meets the requirements of EFSA health claim. Furthermore, the ORAC tests carried out on DES8 (ChCl:citric acid 1 : 1) crude reaction mixture indicate that this mixture, supposed to be biocompatible, could be proposed as ready‐to‐use active formulation able to emulate the antioxidant power of EVOO. Such result is particularly important considering the starting material derivation, such as olive leaves waste of the olive oil supply chain, the simple and environmentally friendly preparation methodology and the improved resistance to oxidation. This last product deserves to be further investigated for its safety for human health and its potential pharmaco‐biological properties.

## Experimental

### Synthesis of DESs

DESs were prepared by heating the components. These were placed in a round‐bottom flask and heated to 80 °C in a water bath with agitation until a homogeneous liquid was formed. Furthermore, 10 % *v/v* water in the DES solutions was added for DES3w‐DES9w.

### General Reaction Conditions

0.358 mmol of oleuropein (193 mg) were added in DES (Oleuropein: Component 1 of DES, 1 : 1 molar ratio). The mixture was heated by microwave assisted or conventional heating (80 °C) for 10–60 minutes. The obtained mixture was analysed by HPLC.

### HPLC Analysis

HPLC analysis was performed using Thermo Scientific (Rodano, MI, Italy) Dionex Ultimate 3000, equipped with a 25 cm ×4.6 mm Thermo Scientific Hypersil GOLD C18 column packed with 5 μm particles. For HPLC separation of the phenolic compounds in DESs, a gradient elution with a mixture of solvents A (H_2_O/trifluoroacetic acid, pH=2.46) and B (methanol) was used. The column was equilibrated in 95 % solvent A and 5 % solvent B. The elution flow rate was 1 mL min^−1^ by linearly increasing of solvent B concentration from 5 to 60 % over 17 min, maintained isocratic for 2 min, subsequently increased to 95 % over 6 min, then returned to 5 % over 3 min and equilibrated for 5 min. The chromatograms were acquired at 280 nm. The instrumentation performance, chromatograms, and initial data processing were carried out with Chromeleon software.

A calibration curve was built using standard solutions of pure oleuropein (10000 ppm), its aglycone form (3,4‐DHPEA‐EA) (10000 ppm) and oleacein ((3,4‐DHPEA‐EDA) (10000) in EtOH; these solutions were then mixed to obtain six standard solutions of 10 ppm, 25 ppm, 50 ppm, 75 ppm, 100 ppm and 125 ppm in both of phenolic compounds. HPLC analysis gave rise to five regression curves (see Supplementary Material). Each DES mixture was diluted in ethanol, which is a miscible solvent in all the deep eutectic solvents used in this study, to obtained solutions of 100 ppm. 20 μL of the diluted extracts were analysed by HPLC and peaks in the chromatograms were identified and quantified by comparison with standards..

### Statistical Analysis

The results were expressed by mean±S.E.M. from at least three independent experiments. They were compared with results reported in Ref 27b as positive control and statistically evaluated for differences by two‐way ANOVA followed by Dunnet test multiple comparison (Prism scientific software).

### Isolation of Synthetic Phenols from DESs: Preparation of SE1 and SE2 Samples

DES5 (CH, 80 °C, 30 min) and DES8 (MW, 149 °C, 60 min) reaction mix (200 mg) were passed through an Amberlyst A21 free base adsorbent filled column with 2.3 cm inside diameter to give a bed height of 6 cm. The adsorbent was pretreated with 20 mL of ethanol and washed with 20 mL of MQ water prior to loading of the phenolic extracts. Extracts were washed with 80 mL of MQ water, and the captured phenols eluted from the resin with 100 mL of ethanol 100 %. Eluted samples were dried under vacuum at 30 °C. HPLC analysis of the mixture is showed in Figure S6 and Figure S7 in the SI file.

### Isolation of Natural Phenols from EVOO: Preparation of NE Sample

EVOO oil produced in 2024 from *Carolea* cultivar was used as starting material.

Natural extract NE was obtained by hydroalcoholic extraction of 5 g of EVOO, and characterized to assess its adhesion to the EFSA claim (Total Organic Phenolic content: 993.81 mg/Kg) as previously described by Frisina et al.^[49]^

## Conflict of Interests

The authors declare no conflict of interest.

1

## Supporting information

As a service to our authors and readers, this journal provides supporting information supplied by the authors. Such materials are peer reviewed and may be re‐organized for online delivery, but are not copy‐edited or typeset. Technical support issues arising from supporting information (other than missing files) should be addressed to the authors.

Supporting Information

## Data Availability

The data that support the findings of this study are available in the supplementary material of this article.
