# Supplementary material for: Environmentally Sustainable Preparation of Oleuropein and Its Dialdehydic Derivatives in a Simple Pharmaceutical Formulation
Source: ChemSusChem. 2025 Jan 7;18(9):e202402252. doi: 10.1002/cssc.202402252 (PMC12051248; doi:10.1002/cssc.202402252)
Supplement: Supplementary file 1 — Supporting Information [file CSSC-18-e202402252-s001.pdf]

ChemSusChem

Supporting Information

Environmentally Sustainable Preparation of Oleuropein and Its Dialdehydic Derivatives in a Simple Pharmaceutical Formulation

Monica Nardi, Sonia Bonacci, Marialaura Frisina, Manuela Oliverio,* Rosa Scarpelli, and Antonio Procopio

Supporting Informations

Environmentally Sustainable Preparation of Oleuropein and Its Dialdehydic Derivatives in a Simple Pharmaceutical Formulation

Monica Nardi,[a] Sonia Bonacci,[a] Marialaura Frisina, [a] Manuela Oliverio,*[a] Rosa Scarpelli,[a] and Antonio Procopio[a]

[a] Department of Health Sciences, University Magna Græcia of Catanzaro, Campus S. Venuta, Viale Europa, Loc. Germaneto, 88100 Catanzaro (Italy).

Index

HPLC analysis

Table S1: Quantitative analysis of Oleacein, Oleuropein Aglycon and Oleuropein in DES mixtures by HPLC p. 3

Figure S1: HPLC linear plot of (a) oleuropein concentration vs area. R2 =0,99989; (b) oleuropein aglycone (3,4-DHPEA-EA) concentration vs area. R2 =0,99911; (c) oleacein (3,4-DHPEA-EDA) concentration vs area. R2 =0,99943. p. 6

Figure S2: HPLC of mix DES8 p. 7

Figure S3: HPLC of mix DES8 after 6 months p. 7

ORAC test

Measurement of oxygen radical absorbance capacity (ORAC) p. 8

Figure S4: FL fluorescence decay curve induced by AAPH p. 9

Figure S5: Linear plot of AUC *vs* Trolox concentrations p. 9

Figure S6: HPLC analysis of SE1 (phenols isolated from DES8) p.10

Figure S7: HPLC analysis of SE2 (phenols isolated from DES5) p.10

Table S1

Table S1: Quantitative analysis of Oleacein, Oleuropein Aglycon and Oleuropein in DES mixtures by HPLC

	Oleacein (ppm)	Aglycon Oleuropein (ppm)	Oleuropein (ppm)	Total ppm	Oleacein (%)	Aglycon Oleuropein (%)	Oleuropein (%)	Conversion (%)
**MW assisted, 149° C, 10 min**								
**DES1**	9.1963 ± 0.13	35.7538 ± 0.65	100.348 ± 2.66	145.2981 ± 1.15	6.3293	24.6072	69.0635	30.9365
**DES2**	243.6991 ± 4.54	774.0286 ± 2.03	1862.1172 ± 6.90	2879.8449 ± 4.49	8.4622	26.8774	64.6603	35.3397
**DES3**	5.8791 ± 0.14	20.7029 ± 1.13	58.3776 ± 1.46	84.9596 ± 0.91	6.9199	24.3680	68.7122	31.2878
**DES4**	7.6579 ± 0.62	17.2755 ± 0.29	54.2079 ± 2.88	79.1413 ± 1.26	9.6762	21.8287	68.4951	31.5049
**DES5**				***carbonized***				
**DES6**	2.6658 ± 0.23	41.2693 ± 2.03	98.2269 ± 8.66	142.1620± 3.64	1.8752	29.0298	69.0950	30.9049
**DES7**	4.3645 ± 0.21	8.6568 ± 0.23	70.2254 ± 8.09	83.2467± 3.03	5.2428	10.3990	84.3582	15.6418
**DES8**	1.4700 ± 0.41	19.8400 ± 0.34	28.2070 ± 2.98	49.5170± 1.24	2.9687	40.0670	56.9643	43.0357
**DES9**	10.8194 ± 0.23	33.8901 ± 1.93	205.0578 ± 2.55	249.7673± 1.57	4.3317	13.5687	82.0995	17.9005
**DES3w**	7.0791 ± 0.93	23.2917 ± 0.84	75.2713 ± 3.76	105.6421 ± 1.84	6.7010	22.0477	71.2512	28.7488
**DES4w**	5.1681± 0.93	17.4253 ± 0.33	52.4361 ± 2.46	75.0295 ± 1.24	6.8881	23.2246	69.8873	30.1127
**DES5w**				***carbonized***				
**DES6w**	0.8791± 0.45	7.0029 ± 0.21	73.3676 ± 4.55	81.2496± 2.66	1.08197	8.6190	90.2990	9.7010
**DES7w**	0.2325± 0.77	3.4529 ± 0.81	76.1096 ± 4.1	79.7950± 1.89	0.2914	4.3272	95.3814	4.6186
**DES8w**	5.3965± 0.33	11.4578 ± 0.77	58.7349 ± 3.76	75.5892± 1.62	7.13925	15.1580	77.7028	22.2972
**DES9w**	9.9964± 2,13	11.2398 ± 0.36	45.3781 ± 3.40	66.6143± 1.96	15.0064	16.8729	68.1206	31.8793
**MW assisted, 149° C, 30 min**								
**DES1**	8.9430 ± 0.13	23.1310 ± 1.09	60.6338 ± 4.31	92.7078 ± 1.84	9.6464	24.9504	65.4031	34.5969
**DES2**	9.4862 ± 0.31	28.1243 ± 0.91	62.4841 ± 6.24	100.0946 ± 2.49	9.4772	28.0977	62.4250	37.5749
**DES3**	7.8539 ± 0.66	25.6509 ± 0.80	61.7786 ± 5.98	95.2834 ± 2.48	8.2427	26.9206	64.8367	35.1633
**DES4**	7.7507 ± 0.21	20.4319 ± 1.89	52.6771 ± 4.92	80.8597 ± 2.34	9.5854	25.2683	65.1463	34.8537
**DES5**				***carbonized***				
**DES6**	1.8590 ± 0.30	46.0286 ± 1.34	92.1172 ± 6.47	140.0048 ± 2.70	1.3278	32.8764	65.7957	34.2042
**DES7**	6.5199 ± 0.51	59.7185 ± 1.76	46.6275 ± 3.22	112.8659 ± 1.83	5.7767	52.9110	41.3123	58.6877
**DES8**	6.9299 ± 0.99	129.4165 ± 3.33	94.1715 ± 2.13	230.5179 ± 2.15	3.0062	56.1416	40.8521	59.1478
**DES9**	17.3497 ± 0.66	158.3493 ± 4.33	120.1329 ± 5.30	295.8319 ± 3.43	5.8647	53.5268	40.6085	59.3915
**DES3w**	4.3390 ± 1.98	18.7941 ± 2.03	42.8471 ± 1.38	65.9802 ± 1.79	6.5762	28.4845	64.9393	35.0607
**DES4w**	8.6971 ± 2.56	33.7939 ± 4.33	82.5536 ± 2.90	125.0446 ± 3.26	6.9552	27.0255	66.0193	33.9807
**DES5w**				***carbonized***				
**DES6w**	41.9090 ± 2.30	117.3054 ± 3.09	655.1878 ± 6.26	814.4022 ± 3.88	5.1460	14.4039	80.4501	19.5498
**DES7w**	17.2925 ± 1.22	101.4592± 3.48	148.1096± 4.03	266.8613 ± 2.91	6.4799	38.0194	55.5006	44.4994
**DES8w**	9.3965 ± 2.33	13.4578± 2.54	61.7349± 4.26	84.5892 ± 3.04	11.1084	15.9096	72.9820	27.0180
**DES9w**	11.9964± 2.95	14.2398± 2.46	45.3781± 2.03	71.6143 ± 2.48	16.7514	19.8840	63.3646	36.6354

MW assisted, 149° C, 60 min

**DES1**	6.7924 ± 0.53	28.6610 ± 0.50	60.3835 ± 2.91	95.8369 ± 1.31	7.0874	29.90601	63.00653	36.9934
**DES2**	9.8165 ± 1.33	33.2435 ± 2.89	77.7214 ± 2.03	120.7814 ± 2.08	8.1275	27.5237	64.3488	35.6511
**DES3**	5.0772 ± 0.13	19.8393 ± 0.39	51.1939 ± 0.65	76.1104 ± 0.39	6.6708	26.0665	67.2627	32.7373
**DES4**	9.1337 ± 1.29	28.4065 ± 1.22	66.2181 ± 2.33	103.7583 ± 1.61	8.8029	27.3776	63.8196	36.1804
**DES5**				***carbonized***				
**DES6**	96.9164± 2.09	388.7285 ± 3.33	903.8588± 6.95	1389.5037± 4.12	6.9749	27.9761	65.0490	34.9510
**DES7**	15.2646± 0.98	150.8871 ± 7.33	103.4805 ± 2.95	269.6322 ± 3.75	5.6613	55.9603	38.37839101	61.6216
**DES8**	16.9299± 0.90	209.4165 ± 2.47	134.1723 ± 6.43	360.5187 ± 3.73	4.6960	58.0876	37.2164	62.7835
**DES9**	14.3756± 1.98	148.7653 ± 2.33	110.1249 ± 2.98	273.2658 ± 2.43	5.2607	54.4398	40.2995	59.7004
**DES3w**	10.1547 ± 0.33	41.2375 ± 2.30	75.8376 ± 2.74	127.2298 ± 1.79	7.9814	32.4118	59.6068	40.3932
**DES4w**	7.8904± 1.96	33.9421± 2.23	63.8321± 2.09	105.6646 ± 2.09	7.4674	32.1225	60.4101	39.5899
**DES5w**				***carbonized***				
**DES6w**	33.6872 ± 3.55	197.4669 ± 7.03	543.4787± 7.33	774.6328 ± 5.97	4.3488	25.4916	70.1595	29.8405
**DES7w**	3.1932 ± 2.33	15.8599 ± 2.92	23.1246 ± 2.93	42.1777 ± 2.73	7.5708	37.6026	54.8266	45.1734
**DES8w**	9.3965 ± 0.39	13.4578 ± 2.53	58.7349± 2.04	81.5892 ± 1.65	11.5168	16.4946	71.9886	28.0114
**DES9w**	11.9964 ± 0.74	14.2398 ± 1.09	45.3781± 4.05	71.6143 ± 1.96	16.7514	19.8840	63.3646	36.6354

Conventional heating, 80° C, 30 min

**DES1**	7.2013 ± 0.33	16.8638 ± 0.90	64.0409 ± 2.98	88.1060 ± 1.40	8.1734	19.1404	72.6862	27.3138
**DES2**	13.0075 ± 0.99	26.5463 ± 2.18	104.2902 ± 4.30	143.8440 ± 2.49	9.0428	18.4549	72.5023	27.4977
**DES3**	9.4344 ± 2.35	18.8884 ± 1.90	67.7666 ± 2.03	96.0894 ± 2.09	9.8183	19.6571	70.5245	29.4755
**DES4**	6.4833 ± 2.12	15.5363 ± 1.85	53.4921 ± 2.43	75.5117 ± 2.13	8.5858	20.5747	70.8395	29.1605
**DES5**	14.4132 ± 2.33	38.1618 ± 3.80	10.75702 ± 1.03	63.3320 ± 2.39	22.7582	60.2567	16.9851	83.0149
**DES6**	78.6040 ± 2.19	102.4586 ± 2.10	1148.7459 ± 6.26	1329.8085 ± 3.52	5.9109	7.7048	86.3843	13.6157
**DES7**	13.873± 2.90	22.6276 ± 2.92	74.1092 ± 2.21	110.6098 ± 2.18	12.5423	20.4571	67.0006	32.9994
**DES8**	9.7890± 2.33	15.0286 ± 0.53	68.1172 ± 3.04	92.9348 ± 2.67	10.5333	16.1711	73.2957	26.7043
**DES9**	10.8194± 2.13	33.8901 ± 4.31	205.0578 ± 2.05	249.7673 ± 2.83	4.3318	13.5687	82.0995	17.9005
**DES3w**	10.9787 ± 0.93	32.2789 ± 1.32	96.2622 ± 1.50	139.5198 ± 1.25	7.8689	23.1357	68.9954	31.0046
**DES4w**	7.4433 ± 0.99	21.8771± 0.61	70.5004 ± 1.42	99.8208 ± 1.01	7.4567	21.9164	70.6270	29.3730
**DES5w**	7.4132 ± 1.03	28.1618± 1.39	9.9570 ± 0.52	45.5320 ± 0.98	16.2813	61.8506	21.8681	78.1319
**DES6w**	41.9090 ± 1.96	117.3054± 3.42	655.1878 ± 1.72	814.4022 ± 2.37	5.1460	14.4039	80.4501	19.5498
**DES7w**	6.0391 ± 0.90	36.5924± 2.70	55.1515 ± 1.20	97.7830 ± 1.60	6.1760	37.4220	56.4019	43.5981
**DES8w**	56.4440 ± 2.76	926.4590± 4.92	696.7939 ± 3.38	1679.6969 ± 3.69	3.3604	55.1563	41.4833	58.5171
**DES9w**	144.0143 ± 9.03	474.6432 ± 4.60	2422.1601 ± 4.39	3040.8176 ± 6.01	4.7360	15.6091	79.6549	20.3451

Conventional heating, 80° C, 60 min

**DES1**	7.4520 ± 0.05	18.4899 ± 1.09	61.7373± 2.42	87.6792± 1.19	8.4992	21.0881	70.4127	29.5873
**DES2**	6.9496 ± 0.23	18.8590 ± 1.08	54.8067± 1.19	80.6153± 1.49	8.6207	23.3938	67.9855	32.0145
**DES3**	8.4265 ± 1.35	23.0956 ± 1.43	70.1515 ± 2.09	101.6736± 1.62	8.2878	22.7154	68.9968	31.0032
**DES4**	7.0161 ± 2.89	18.8989 ± 1.07	70.1515 ± 2.55	96.0665± 2.17	7.3034	19.6727	73.0239	26.9761
**DES5**	15.8877 ± 1.95	41.5624 ± 2.32	50.1515 ± 2.98	107.6016± 2.42	14.7657	38.6262	46.6085	53.3914

**DES6**	16.5823 ± 1.06	89.0458 ± 2.05	125.2356 ± 2.34	230.8637± 1.82	7.1827	38.5707	54.2466	45.7534
**DES7**	7.9013 ± 0.45	21.0938 ± 1.09	37.1789 ± 2.30	66.1740± 1.28	11.9402	31.8763	56.1835	43.8165
**DES8**	18.7391 ± 2.05	50.0193 ± 1.43	73.9813 ± 5.05	142.7397± 2.84	13.1283	35.0423	51.8295	48.170
**DES9**	9.6858 ± 0.45	41.5624 ± 2.45	70.1515 ± 1.95	121.3997± 1.62	7.9784	34.2360	57.7856	42.2144
**DES3w**	11.9164 ± 0.33	281.7285 ± 0.53	1103.8588 ± 5.01	1397.5037 ± 1.96	0.8527	20.1594	78.9879	21.0121
**DES4w**	18.9360 ± 0.12	30.1380 ± 2.93	70.1092 ± 1.44	119.1832 ± 1.49	15.8881	25.28712	58.8247	41.1753
**DES5w**	9.6858 ± 0.30	41.5624 ± 1.22	70.1515 ± 0.44	121.3997 ± 0.65	7.9784	34.2360	57.7856	42.2144
**DES6w**	33.6872 ± 0.62	217.4669 ± 1.03	443.4781± 0.24	694.6328 ± 0.63	4.8496	31.3067	63.8436	36.1564
**DES7w**	7.3170 ± 0.24	20.4430 ± 0.70	38.6000 ± 0.22	66.3600 ± 0.39	11.0262	30.8062	58.1676	41.8324
**DES8w**	13.6858 ± 0.11	40.5754 ± 0.22	68.1455 ± 0.55	122.4067 ± 0.29	11.1806	33.1480	55.6714	44.3286
**DES9w**	9.6858 ± 0.23	41.5624 ± 0.22	79.1928 ± 0.13	130.4410 ± 0.19	7.4254	31.8630	60.7116	39.2884

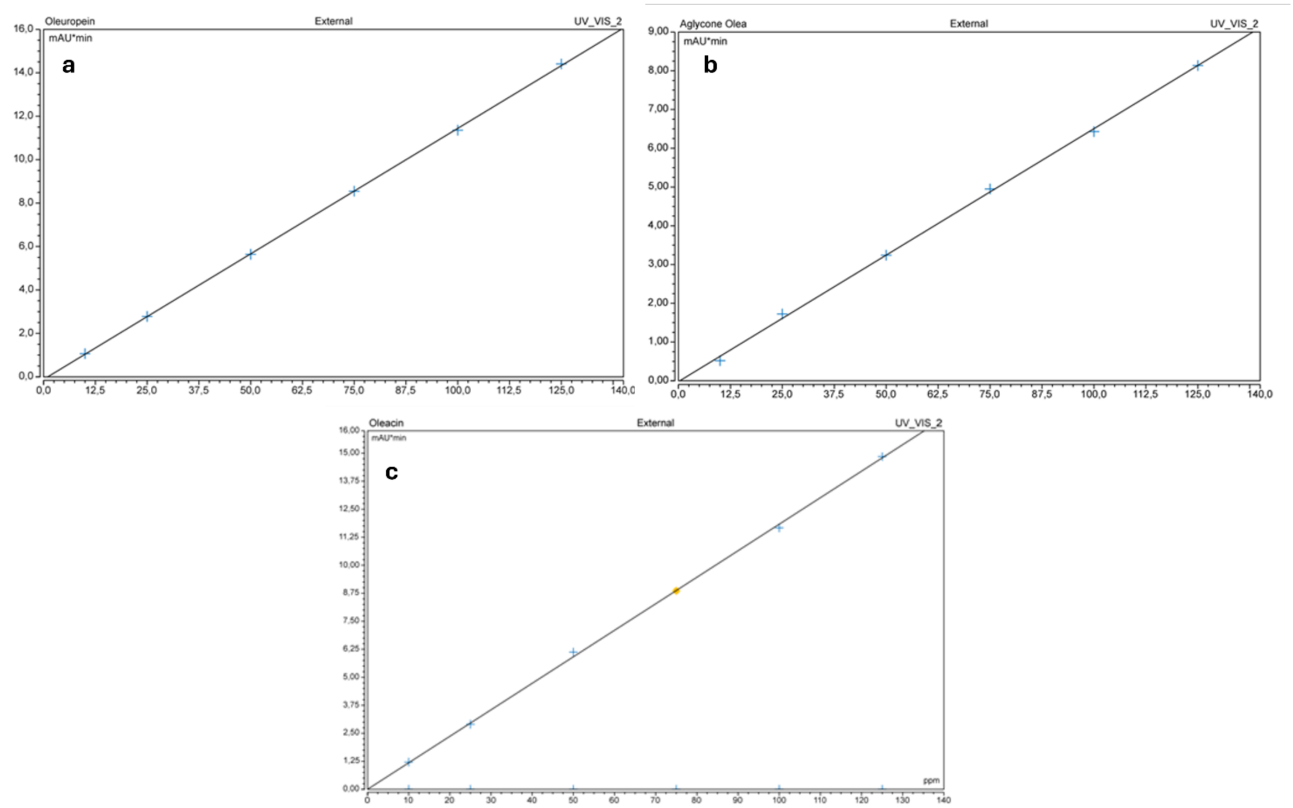

Figure S1: HPLC linear plot of (a) oleuropein concentration vs area. R2 =0,99989; (b) oleuropein aglycone (3,4-DHPEA-EA) concentration vs area. R2 =0,99911; (c) oleacein (3,4-DHPEA-EDA) concentration vs area. R2 =0,99943.

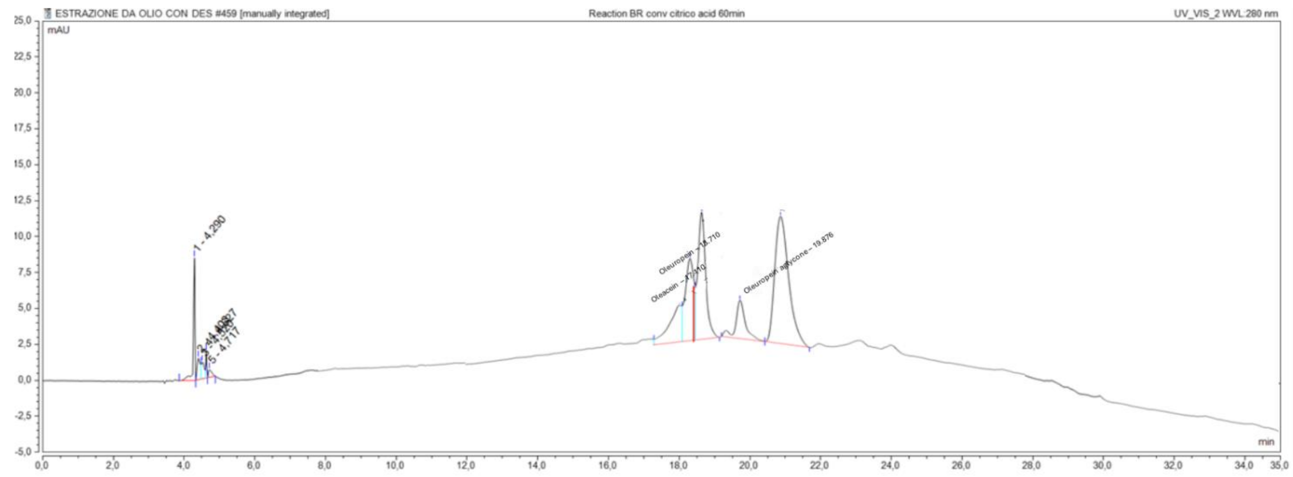

Figure S2: HPLC of mix DES8

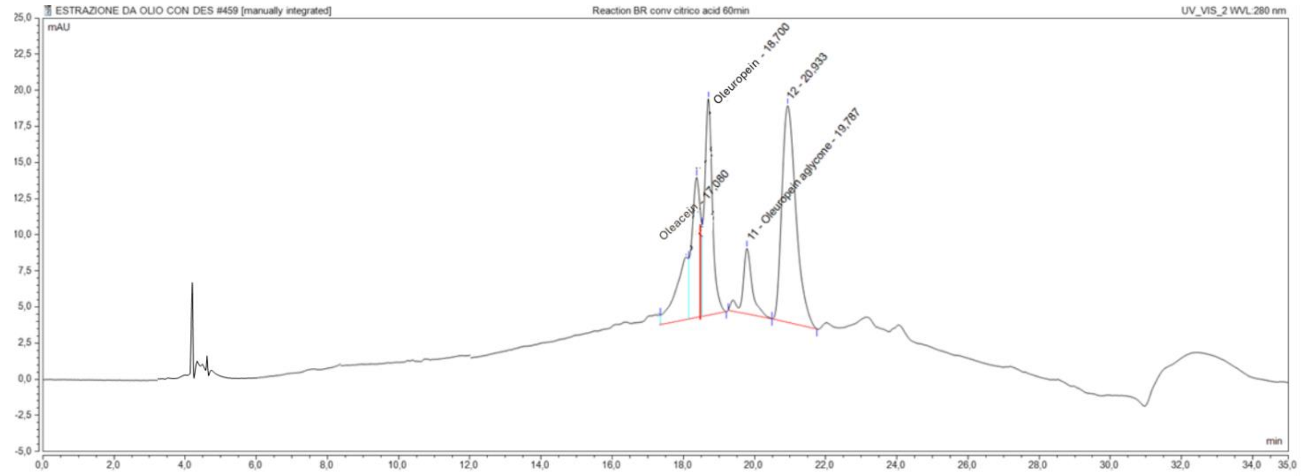

Figure S3: HPLC of mix DES8 after 6 months

ORAC tests

Measurement of oxygen radical absorbance capacity (ORAC)

The ORAC assay employs a microplate fluorometer Varioskan LUX (Thermo Scientific™), controlled by Thermo Scientific™ SkanIt™ Software for microplate readers. Fluorescence (excitation 495 nm; emission, 518 nm) was measured in a kinetic reaction for 90 minutes, with an interval of 1 minute, using a 96-well plate. A 10 mM PBS solution (pH= 7.4) was used as blank. 5 mg L^{-1} solutions of selected reaction mixtures and natural EVOO extract in PBS were used as samples. To each well 16 μl of sample or blank or Trolox (6.25, 12.5, 25, 50 and 100 μM in PBS) were added to 160 μl of disodium fluorescein solution (FL, 14 μM in PBS, pre-incubated at 37°C for 15 min). Each solution was analyzed in duplicate in a “forward-then-reverse” order as described in the literature [Prior R. L. et al., J. Agric. Food Chem., 2003, 51, 3273-3279. DOI: 10.1021/jf0262256]. Reactions were initiated by the addiction of 60 μl of AAPH (2,2'-azobis(2-amidino-propane) dihydrochloride, 31.7 mM in PBS). Measurement temperature was set at 37°C. The final $ORAC_{FL}$ value were calculated using a regression equation between the Trolox concentration and the net area under the FL decay curve [Prior R. L. et al., J. Agric. Food Chem., 2005, 53, 4290-4302 DOI:10.1021/jf0502698]:

$$Y(\mu M\ Trolox) = a + bX(AUC\_n)$$

and were expressed as Trolox equivalents (μmol gr^{-1}). The area under the curve was calculated as:

$$AUC = 1 + \frac{f_1}{f_0} + \frac{f_2}{f_0} + \frac{f_3}{f_0} + \cdots . + \frac{f_{45}}{f_0}$$

Where f_0 is the initial fluorescence reading, f_i is the reading at the cycle i.

The net AUC was obtained as AUC_{sample}-AUC_{blank}.

Figure S4: FL fluorescence decay curve induced by AAPH.

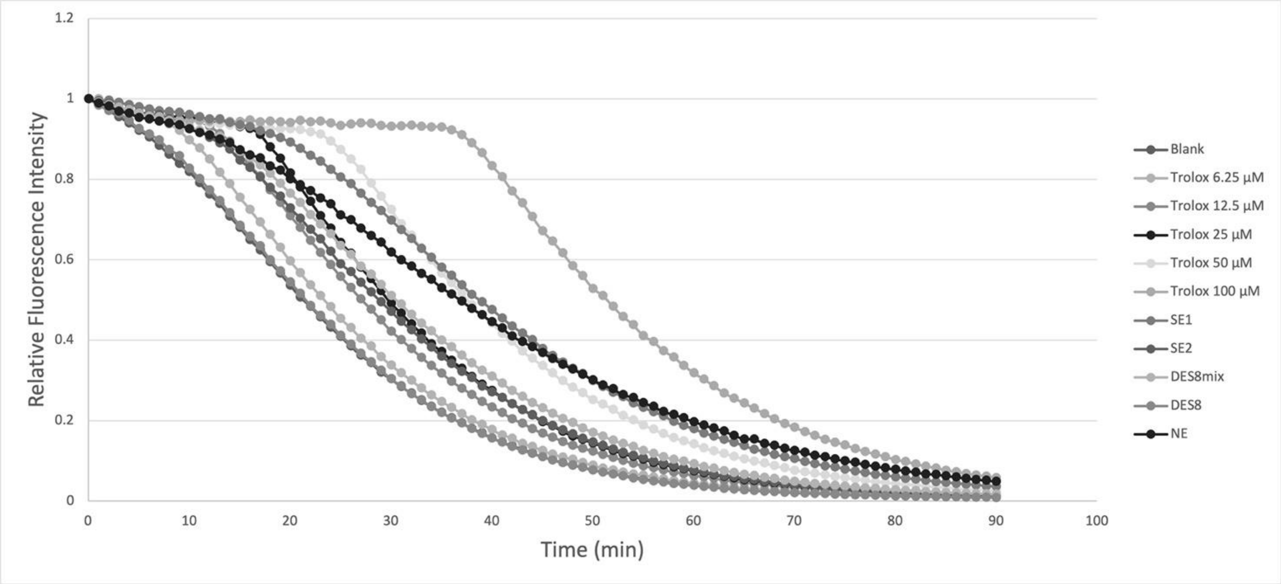

Figure S5: Linear plot of AUC *vs* Trolox concentrations.

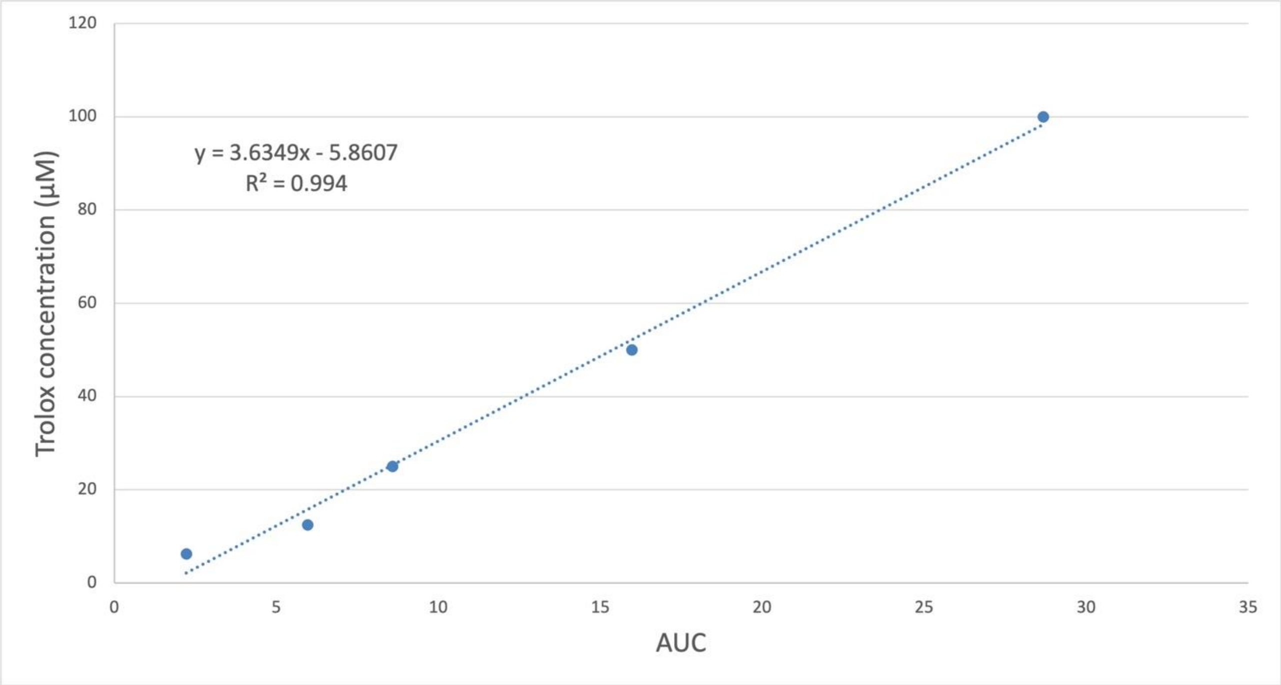

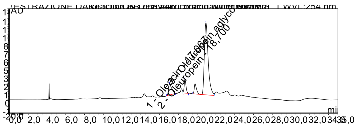

Figure S6: HPLC analysis of SE1 (phenols isolated from DES8)

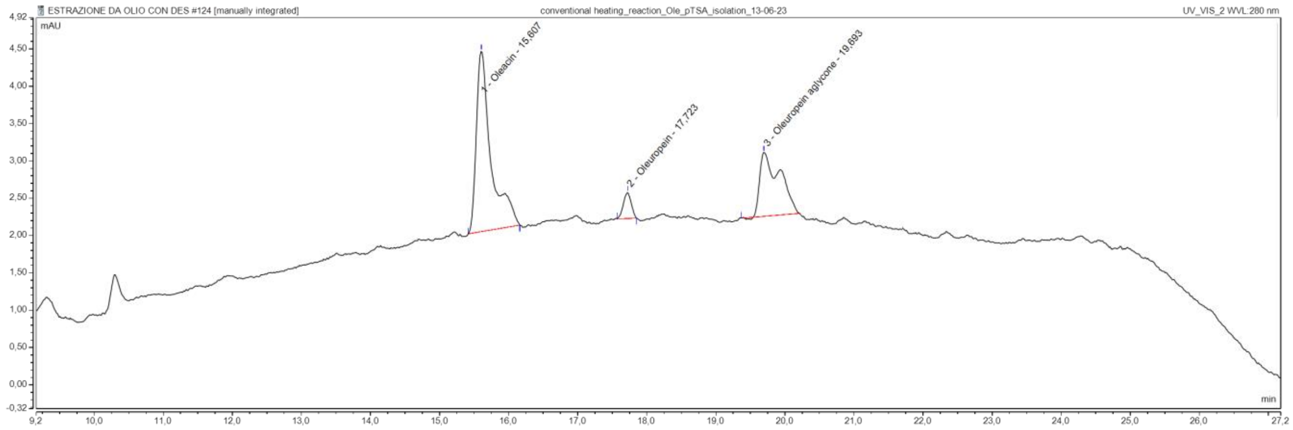

Figure S7: HPLC analysis of SE2 (phenols isolated from DES5)
